# Identification of AIDS-Associated Kaposi Sarcoma: A Functional Genomics Approach

**DOI:** 10.3389/fgene.2019.01376

**Published:** 2020-01-24

**Authors:** Peng Zhang, Jiafeng Wang, Xiao Zhang, Xiaolan Wang, Liying Jiang, Xuefeng Gu

**Affiliations:** ^1^School of Clinical Medicine, Shanghai University of Medicine & Health Sciences, Shanghai, China; ^2^Department of Public Health, Shanghai General Practice Medical Education and Research Center, Shanghai, China; ^3^Stem Cell Research and Cellular Therapy Center, Affiliated Hospital of Guangdong Medical University, Zhanjiang, China; ^4^Department of Implant Dentistry, Ninth People's Hospital Affiliated to Shanghai Jiaotong University School of Medicine, Shanghai, China; ^5^College of Nursing and Health Management, Shanghai University of Medicine & Health Sciences, Shanghai, China; ^6^Shanghai Key Laboratory of Molecular Imaging, Collaborative Research Center, Shanghai University of Medicine & Health Sciences, Shanghai, China

**Keywords:** herpesvirus, immune evasion, sequence homology, protein–protein interactions, AIDS, ORF-73

## Abstract

**Background:**

Kaposi sarcoma-associated herpes virus (KSHV) is one of the most common causal agents of Kaposi Sarcoma (KS) in individuals with HIV-infections. The virus has gained attention over the past few decades due to its remarkable pathogenic mechanisms. A group of genes, ORF71, ORF72, and ORF73, are expressed as polycistronic mRNAs and the functions of ORF71 and ORF72 in KSHV are already reported in the literature. However, the function of ORF73 has remained a mystery. The aim of this study is to conduct comprehensive exploratory experiments to clarify the role of ORF73 in KSHV pathology and discover markers of AIDS-associated KSHV-induced KS by bioinformatic approaches.

**Methods and Results:**

We searched for homologues of ORF-73 and attempted to predict protein-protein interactions (PPI) based on GeneCards and UniProtKB, utilizing Position-Specific Iterated BLAST (PSI-BLAST). We applied Gene Ontology (GO) and KEGG pathway analyses to identify highly conserved regions between ORF-73 and p53to help us identify potential markers with predominant hits and interactions in the KEGG pathway associated with host apoptosis and cell arrest. The protein p53 is selected because it is an important tumor suppressor antigen. To identify the potential roles of the candidate markers at the molecular level, we used PSIPRED keeping the conserved domains as the major parameters to predict secondary structures. We based the FUGE interpretation consolidations of the sequence-structure comparisons on distance homology, where the score for the amino acids matching the insertion/deletion (indels) detected were based on structures compared to the FUGE database of structural profiles. We also calculated the compatibility scores of sequence alignments accordingly. Based on the PSI-BLAST homologues, we checked the disordered structures predicted using PSI-Pred and DISO-Pred for developing a hidden Markov model (HMM). We further applied these HMMs models based on the alignment of constructed 3D models between the known structure and the HMM of our sequence. Moreover, stable homology and structurally conserved domains confirmed that ORF-73 maybe an important prognostic marker for AIDS-associated KS.

**Conclusion:**

Collectively, similar variants of ORF-73 markers involved in the immune response may interact with targeted host proteins as predicted by our computational analysis. This work also suggests the existence of potential conformational changes that need to be further explored to help elucidate the role of immune signaling during KS towards the development of therapeutic applications.

## Introduction

Pre-existing human immunodeficiency virus (HIV) infections affect the immune system increasing the risk for development of Kaposi sarcoma (KS). Since the discovery of Kaposi sarcoma-associated herpesvirus (KSHV), also termed human herpesvirus 8 (HHV8), the tumor development and oncogenesis were associated with co-expression of different genes (Barré-Sinoussi et al., 1983; Gelmann et al., 1983). KS is a common type of cancer associated with blood vessels and lymph nodes. Soon after the discovery of HIV-1, scientists discovered γ-herpesvirus in KS lesions (Chang et al., 1994). Now that the full KSHV genome has been sequenced, it fulfils Koch's modern postulates linking the KS cancer initiation to the oncogenic virus (Russo et al., 1996; zur Hausen, 2001). KSHV is a key viral pathogen in cancer biology affecting humans and its discovery promoted clinical and epidemiological research into viral oncology (Chang et al., 1994). However, many questions remain unanswered due to the significant mortality and rapid morbidity of those affected by HIV-1 and KSHV (Parkin, 2006; Sinfield et al., 2007; Dittmer and Damania, 2019; Gaur et al., 2019).

In fact, KS was named after Dr. Moritz Kaposi, a prominent Hungarian dermatologist, who described KS as an ‘idiopathic pigmented sarcoma of the skin' in 1872 (Kaposi, 1872). The evolved gamma-herpesviruses have been classified into many subfamilies (Roizman et al., 1981) and produce many viral gene products capable of subverting the normal cellular machinery through processes involving apoptosis, cell cycle progression, antiviral responses, and immune surveillance resulting in alterations in master cell signaling pathways to establish a persistent host infection. The double-stranded KSHV genome (124–174 kb) is enclosed in an icosahedral capsid composed of 162 capsomeres with many of its ORFs being conserved in alpha- and beta-herpesviruses, but absent from other herpesviruses.

The KSHV is closely related to the subfamily Rhadinoviridae (gamma-2-herpesviruses), which is also close to the Herpes virus saimiri (HVS); therefore, similarities between ORFs of KSHV and HVS may influence the pathogenesis of KS (Schäfer et al., 2003). The HVS genome exists as a stable non-integrated circular episome in altered human and simian T cells. A group of genes, ORF71, ORF72, and ORF73, are located at the right end of the L-DNA and are expressed as polycistronic mRNAs (Fickenscher et al., 1996). Initial studies discerned that both KSHV and HVS ORF71 encode the anti-apoptotic FLICE inhibitory protein (vFLIP) (Thome et al., 1997), although HVS ORF71 is not mandatory for viral replication, transformation, or pathogenicity (Glykofrydes et al., 2000). ORF72 produces a v-Cyclin D homolog which is important for transformation of human T lymphocytes (Ensser et al., 2001). However, the function of ORF73 has remained a mystery. Therefore, developing and conducting comprehensive exploratory experiments to clarify the role of ORF73 in KSHV pathology is important.

Typically, the phenotypic features of KS initially appear on the face, legs, or feet as painless red spots but, in severe cases, the lesions also appear in the lungs and digestive tract (Bhutani et al., 2015; Yarchoan et al., 2015). KSHV is considered an oncogenic human virus (Martin et al., 1998). People with weak immune systems are more susceptible to HHV-8 infection (triggering KS development). Even with the availability of the anti-retroviral treatment [HAART], the prevalence of AIDS-associated KS has not declined significantly (Nguyen et al., 2008). Although KSHV infection is important for the onset of KS, additional factors must be present to allow the establishment of the lesions. The chance of infection is one in 100,000 among the general population, but only around one in 20 among HIV-infected individuals (La Ferla et al., 2013). The chance of acquiring the infection was one in three among HIV-infected individuals before the introduction of HAART (Beral et al., 1990; Gallo, 1998). Epidemiological observations from incidence rates in endemic areas suggest that HIV-negative individuals with KSHV infections never develop KS due to the role of immunological host factors including immune-response genes and genetic polymorphisms of the inflammatory modulators (Cottoni et al., 2004; Gazouli et al., 2004; Dorak et al., 2005).

KSHV infection of endothelial and/or hematopoietic progenitors (Della Bella et al., 2008) alter their morphology (Moses et al., 1999), growth rate, gene expression (Flore et al., 1998; Ciufo et al., 2001), and glucose metabolism (Delgado et al., 2010), leading to development of KS. Antibody titers specific for KSHV correlate with its viral load. Among individuals with low viral load, antibody titer concentrations may be too low for current serological assays to identify them. Identification of circulating biomarkers in KSHV-associated disease may help in predicting clinical outcomes (Aka et al., 2015). Immune modulatory and evasion proteins of KSHV modulate cellular responses associated with complement activation, autophagy, IFN family signaling, chemokines, natural killer cells, and apoptosis (Liang et al., 2008). They are located in a region of the viral capsid that is rich in a protein known as tegument. Six tegument proteins have been identified: ORF21, ORF33, ORF45, ORF63, ORF64, ORF73 and ORF75. Among these, the roles of ORF63 and ORF64 in immune evasion have been elucidated (Zhu et al., 2005; Gregory et al., 2011). We focused on the identification of the role of ORF73 in KSHV. The ORF73 gene encodes the HHV-LANA1 viral proteins that have been linked with AIDS-associated KS, indicating an association between HIV and ORF73. For our computational study, we hypothesized that ORF-73 is a viral proliferation factor based on studies on KS and on its interactions with the host gene p53 (Woodberry et al., 2005). The importance of ORF-73 for cellular host apoptosis through the p53 signaling pathway and p53 is in order of ORF-73 which illustrates the molecular mechanism of this key biomarker associated with KS (Duus et al., 2004).

The variability in KS lesions observed in histopathological assays include spindle cell hemangiomas, cutaneous angiosarcomas, vascular leiomyomas, and fibrous histiocytomas (Hunt et al., 2004). Endothelial biomarkers, such as CD31 and CD34, bcl-2, c-kit, Ki-67, and p53, have been used to distinguish nonvascular spindle sarcomas from angiosarcomas (Weeden, 2002; Fukunaga, 2005). Hence, investigating the HHV-latent associated nuclear antigen-1 (LANA-1) viral protein encoded by ORF-73 is important to identify markers for AIDS-associated KS. Also, studying its interactions may help in the development of preventive strategies and therapeutic options against KS. In this study, we used advanced bioinformatics tools and approaches to identify KS markers **Supplementary Figure 1**.

## Materials and Methods

### Selection of Markers

We used publicly available databases including the National Centre for Biotechnology Information (NCBI), GeneCards (Hou et al., 2017) and UniProtKB (Tang et al., 2013) to identify potential markers of KS and selected the most specific ones using “Kaposi's sarcoma” as a keyword. Human protein markers were further ran through a BLAST search for homology sequences. We extracted ORF-73 sequences from the NCBI database search using the accession number AAC57158.1. These are the exact URLs of the searched databases we used to identify markers associated with KS : GeneCards https://genecards.weizmann.ac.il/v3/index.php?path=/Search/keyword/kaposi%20sarcoma%20markers/0/20; UniPortKB https://www.uniprot.org/uniprot/?query=kaposi+sarcoma&sort=score; and NCBI https://www.ncbi.nlm.nih.gov/protein/?term=ORF-73%20kaposi%20sarcoma).

### Bioinformatics: Sequence Computational Analysis

We used publicly available internet-based protein search tools and bioinformatics programs with default settings, unless otherwise stated in the text, for the analysis. We tested selected protein sequences to identify conserved domains from NCBI and BLAST algorithms, and we used the PSIPRED program to predict the secondary structure of proteins based on the conserved domain sequences. We further executed a position specific iterative BLAST (PSI-BLAST) search to build a PSSMs (position specific score matrix), which could predict the secondary structure of the input sequences (Majerciak et al., 2015) to predict secondary structures of the selected conserved domains based on multiple sequence alignment related proteins spanning a variety of organisms to reveal sequence regions containing the same, or similar, patterns of amino acids. We submitted the primary sequence of ORF-73 to FUGUE to show the sequence-structural homology by identifying distant sequence-structure homologues and alignments comparing amino acid insertions/deletions (Shi et al., 2001). We used BLASTp and PSI-BLAST (non-redundant protein databases) for pattern specific profiling (Bujnicki and Rychlewski, 2001).

### Gene Ontology and Pathway Enrichment Analysis

We chose the ORF-73 target effector to perform a Gene Ontology (GO) search, is a hierarchical graph-based annotation system where the terms closer to the root describe more general information while those away from the root provide more specific information about a given GO category and all the GO terms associated with a protein sequence were obtained from the GO database. The KEGG network pathway enrichment analysis by collecting data of related genomes and their pathways associated with diseases (Yan et al., 2013) and we set a *P* value <0.05 as the cut-off criterion.

### Protein–Protein Interaction (PPI) Network Analysis

We used the online Search Tool for the Retrieval of Interacting Genes (STRING) (Franceschini et al., 2013) and GeneMania (https://genemania.org/) to analyze interactions associated with KS among the proteins encoded by the DEGs. The two parts of GeneMania algorithm consists of an algorithm based on linear regression to calculate functional association from multiple networks from different data sources; and a label predicting gene function of composite network. We employed keywords such as—ORF73 to determine interacting partners. This was pursued using downstream regulator p53 as an apoptosis marker during pathogenesis in the host. Moreover, the marker protein was used for transient interaction study.

### PPI Biochemical Analysis

We immobilized His-tag, GST-tag, or biotin-tag bait proteins to an affinity resin and incubated them with solution expressed proteins as prey proteins. We then captured the bound bait and pulled down the cell lysate flow through. Subsequently, we used mass spectrometry (MS) or Western blots to confirm interactions. Using this technique, we determined interacting protein partners of relevant proteins (Einarson, 2001; Arifuzzaman et al., 2006).

## Results

### Homology Search and KS Marker Identification

Annotations used to search for the KS-associated markers in the UniProtKB database quoted about 137 entries, which we then screened to find those with computationally annotated data. Search engine GeneCards reported about 369 KS markers with a relevance score. **Table 1** lists the markers with the top ten scores.

**Table 1:** GeneCards and UniPortKB databases used to choose the top-most scored identities of markers associated with KS.

GeneCard database				
Sl. No	Symbol	Description	GC id	Score
1	KRT15	Keratin 15	GC17M039675	1.58
2	OSM	Oncostatin M	GC22M030658	1.58
3	TAT	Tyrosine aminotransferase	GC16M071599	1.27
4	MKI67	Marker of proliferation Ki-67	GC10M129894	1.14
5	CD34	CD34 molecule	GC01M208057	1.11
6	PTX3	Pentraxin 3, long	GC03P157154	1.09
7	PECAM1	Platelet/endothelial cell adhesion molecule 1	GC17M062399	1.01
8	FLI1	Fli-1 proto-oncogene, ETS transcription factor	GC11P128596	1.01
9	IFNA2	Interferon, alpha 2	GC09M021374	1.01
10	ACTC1	Actin, alpha, cardiac muscle 1	GC15M035080	0.99
**Uniport KB database**				
**Sl. No**.	**Entry name**	**Protein name**	**Entry**	**Gen name**
1	MIR1_HHV8P	E3 ubiquitin-protein ligase MIR1	P90495	K3
2	MIR2_HHV8P	E3 ubiquitin-protein ligase MIR2	P90489	K5
3	GB_HHV8P	Envelope glycoprotein B	F5HB81	gBORF8
4	ARBH_HHV8P	Apoptosis regulator Bcl-2 homolog	F5HGJ3	vBCL2 ORF16
5	SCAF_HHV8P	Capsid scaffolding protein	Q2HRB6	ORF17
6	OX2V_HHV8P	OX-2 membrane glycoprotein homolog	P0C788	K14
7	GN_HHV8P	Envelope glycoprotein N	F5HFQ0	gN ORF53
8	GM_HHV8P	Envelope glycoprotein M	F5HDD0	gM ORF39
9	ORF45_HHV8P	Protein ORF45	F5HDE4	ORF45
10	VMI2_HHV8P	Viral macrophage inflammatory protein	Q98157	ORF K4
11	VIRF1_HHV8P	VIRF-1	F5HF68	vIRF-1
12	ICP27_HHV8P	mRNA export factor ICP27 homolog	Q2HR75	ORF57
13	GH_HHV8P	Envelope glycoprotein H	F5HAK9	gH ORF22
14	AN_HHV8P	Shutoff alkaline exonuclease	Q2HR95	ORF37
15	LANA1_HHV8P	Protein LANA1	Q9QR71	LANA1 ORF73

We found61 ORF-73 marker homologous hits related to the family of human gamma herpes virus 8 with varied E-values. Out of these, we used only the most identical sequence (based on sequence identity was measured by matched by dividing the length of region aligned match), AAC57158.1, for our computational analyses. A search for proteins similar to the selected marker ORF-73 resulted in8 protein accessions (ORF21, ORF33, ORF45, ORF63, ORF64, and ORF75), and 2 CDS regions (accession numbers AAC57158.1 and AAC55944.1).

### Domain Prediction and Structural Profile

**Figure 1:**
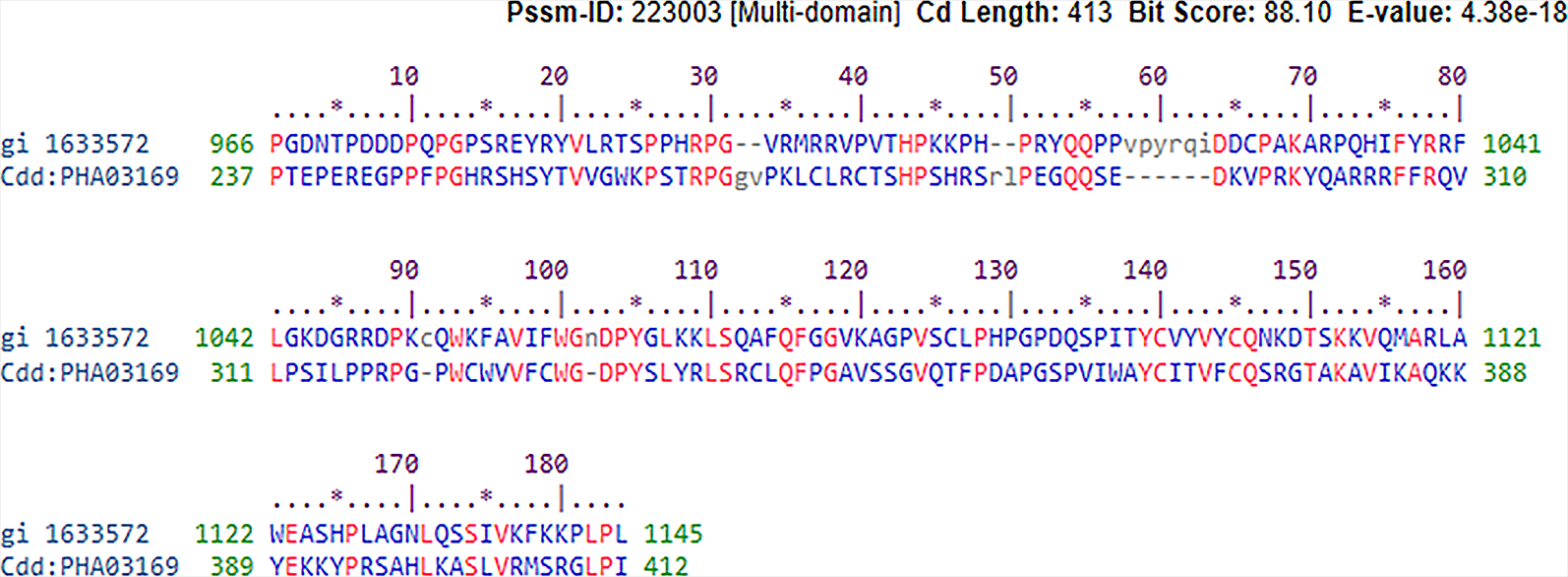
Conserved hypothetical protein domain of PHA03169 in reference to the ORF-73 of Human gamma herpesvirus 8,E-value 38e−18.

**Figure 2:**
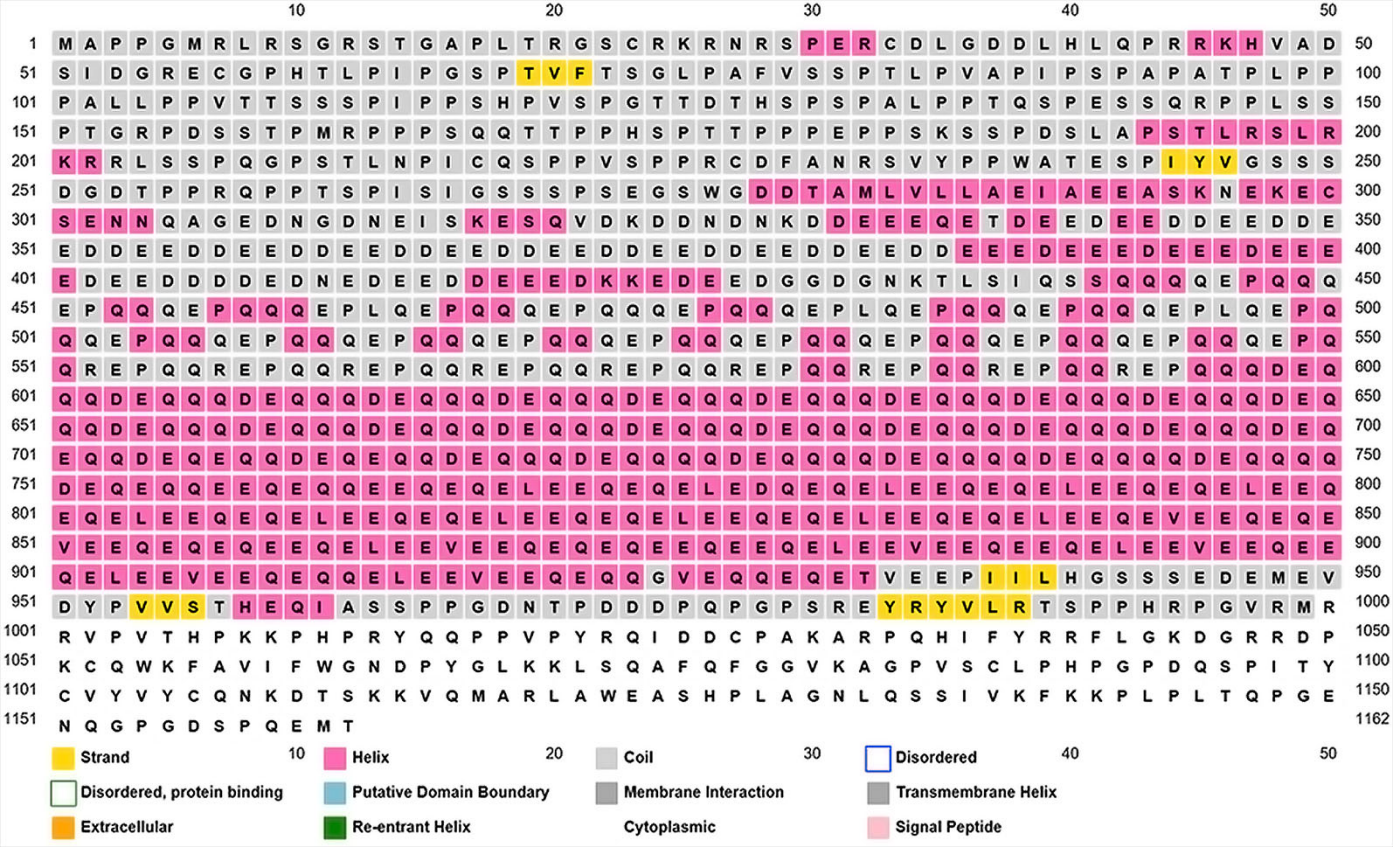
Overview of the ORF-73 secondary structure prediction. The predicted structural positions incorporate two feed-forward neural networks obtained from PSI-BLAST.

We looked for conserved domains in the marker protein ORF-73 based on hypothetical domain sequences using literature recapitulation NCBI's Conserve Domain Database (CDD). To identify potential marker roles at the molecular level, we focused on its predicted secondary structure. Therefore, we searched for hypothetical protein having conserved domain and used accession number AAC5744 of gi.1633572 in an NCBI domain search and found only one significant hypothetical conserved domain (PHA03169) with the same accessison number (**Figure 1**). We then used PSIPRED to predict the secondary structure, noted the conserved domains (**Figure 2**) and highlighted the regions with different markers to predict the secondary structures. FUGE interpretation consolidations of the sequence-structure comparison were based on distance homology, where the score for the amino acids matching the insertion/deletion (indels) detected were based on structures compared to the FUGE database of structural profiles and we calculated the compatibility scores of sequence alignment accordingly (**Table 2**).

**Table 2:** Structure of Kaposi sarcoma marker ORF-73 predicted based on an environmental-specific substitution table and its structure-dependent gap penalties.

Sl. No.	Profile Hit	PLEN	RAWS	RVN	ZSCORE
1	hs4blga	121	−755	247	24.21
2	hs2ap3a	191	215	8	17.29
3	hs2qiha	136	−822	10	16.57
4	hs2p03a	323	249	21	14.78
5	hs1i4da	188	157	33	14.61
6	hs4cgka	351	325	115	13.67
7	hs2eqbb	93	−880	5	13.53
8	hs1fxka	103	168	19	13.45
9	hs1owaa	156	166	6	13.28
10	hs4hpqc	396	−555	5	12.92

PLEN, Profile length; RAWS, Raw alignment score; RVN, (Raw score)-(Raw score for NULL model); ZSCORE, Z-score normalized by sequence divergence (evolutionary relationship associated with a score >5.0 to the sequences are compared to each other); ZORI, Original Z-score (before normalization).

**Figure 3:**
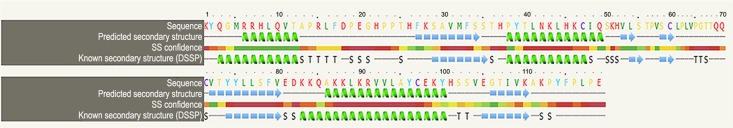
Highest scored template c4k2jB chain B structure.

**Figure 4:**
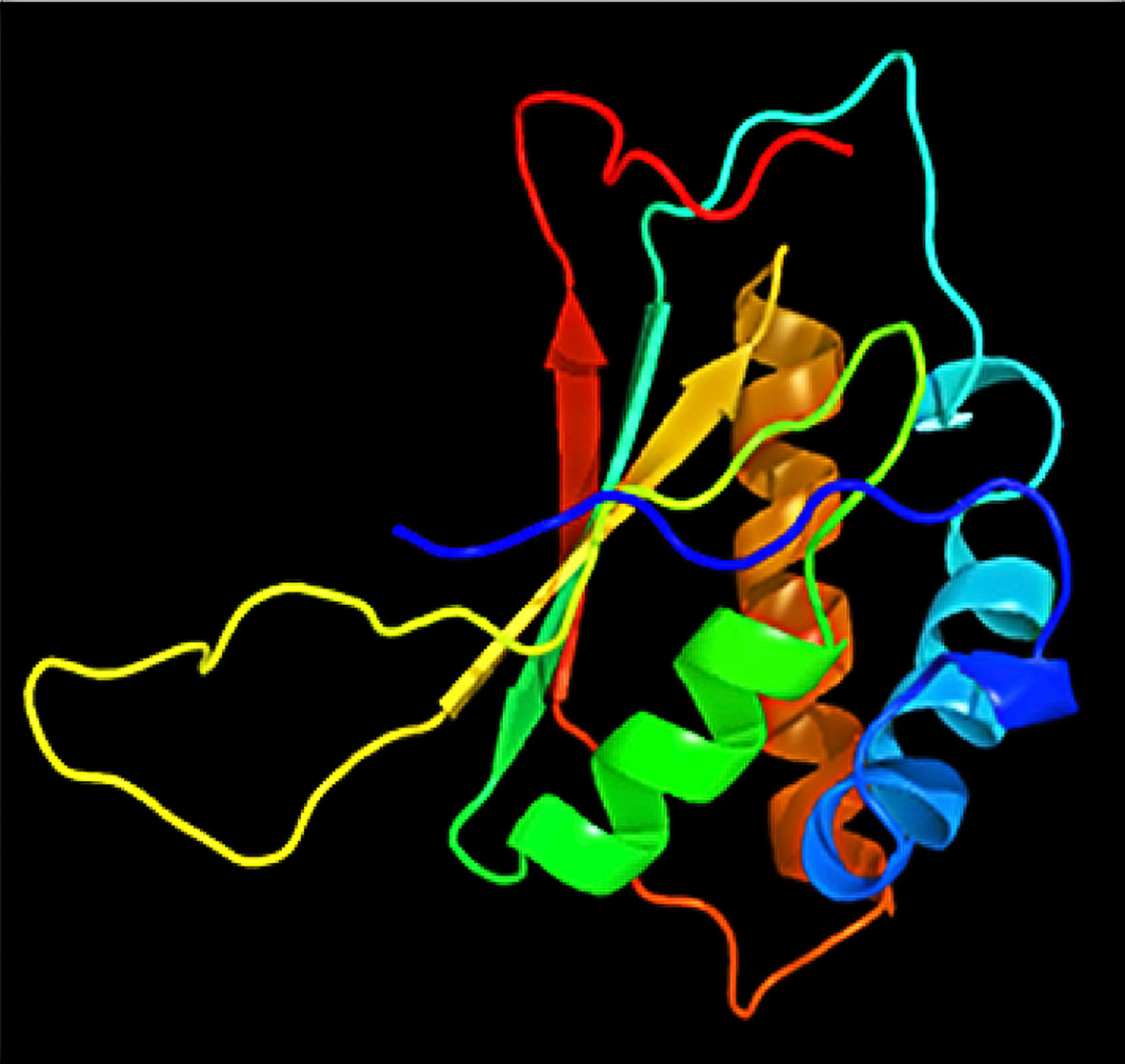
Decameric ring structure of KSHV HHV-LANA DNA binding domain with dimensions (X:40.909, Y:43.389, and Z:44.674).

Using PSI-BLAST, we confined the search of HHV-latency-associated nuclear antigen homology to ORF-73 homologs. The DNA binding of viral protein associated with HHV-8 LANA sheltered 134 residues covering 12% of the sequence with 100% confidence based on the single highest scoring template of c4k2jB (**Figures 3** and **4**). 598 residues covering 51% could be modelled at >90% confidence using multiple-templates. We submitted the top-ranking model of the protein (c4k2jB, 100.0% confidence) to the 3DLigandSite (Wass et al., 2010) server to predict potential binding sites. Based on PSI-BLAST homologues, the predicted disordered structures were checked using PSI-Pred (Jones, 1999) and DISO-Pred (Jones and Cozzetto, 2015) for generating a hidden Markov model (HMM). The models were based on the alignment of the constructed 3D models between the known structure and the HMM of our sequence predicting the3-states—α-helix, β-strand or coil (“SS” indicates the predicted confidence; middle orange, yellow, and green indicate the confidence of prediction).

### Gene Expression and Pathway Prediction

**Figure 5:**
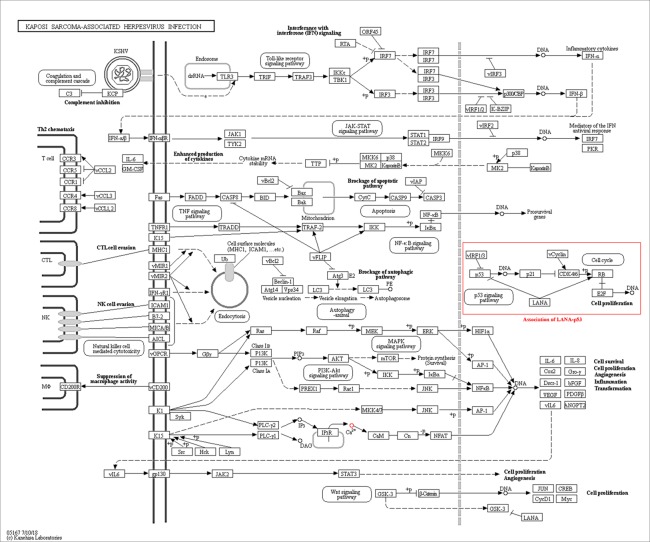
The Kaposi sarcoma-associated herpesvirus infection pathway from KEGG. Reference pathway highlighted using red box shows that LANA is associated with p53 signaling pathway which confirms the predictable role of the ORF-73 protein in the KS associate marker protein.

**Figure 6:**
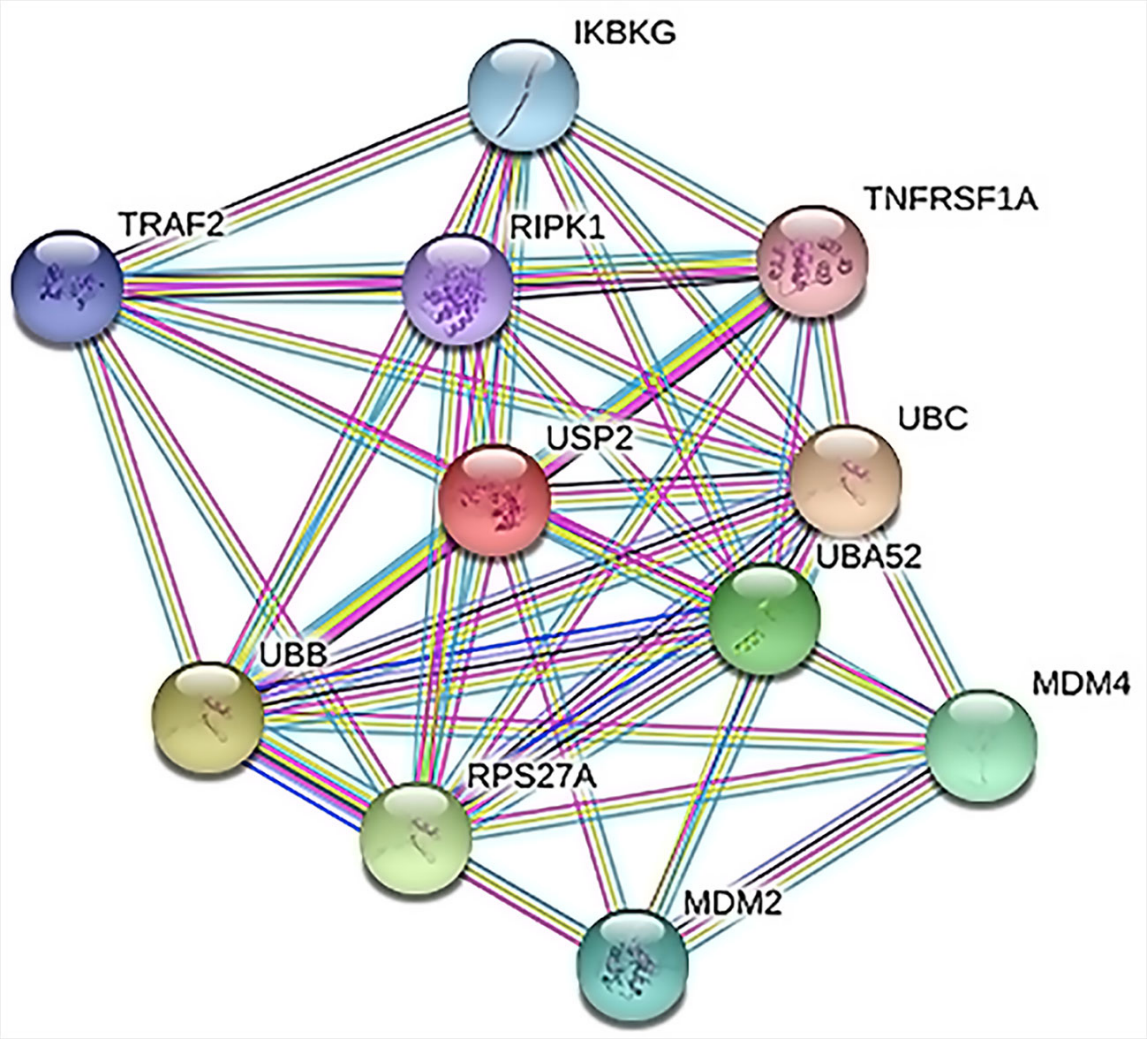
Protein–protein interactions (PPI) between cell arrest marker p53 of cancer cell and Ubiquitin Specific Peptidase 2 (USP2). TNF receptor-associated factor 2 (TRAF2), tumor necrosis factor receptor superfamily member 1A (TNFRSF1A), polyubiquitin-C (UBC), protein Mdm4, E3 ubiquitin-protein ligase Mdm2, ubiquitin-40S ribosomal protein S27a, polyubiquitin-B (UBB), NF-kappa-B essential modulator (IKBKG), receptor-interacting serine/threonine–protein kinase 1 (RIPK1), and Ubiquitin-60S ribosomal protein L40 (UBA52) play important roles in the regulation of cell survival and apoptosis.

**Figure 7:**
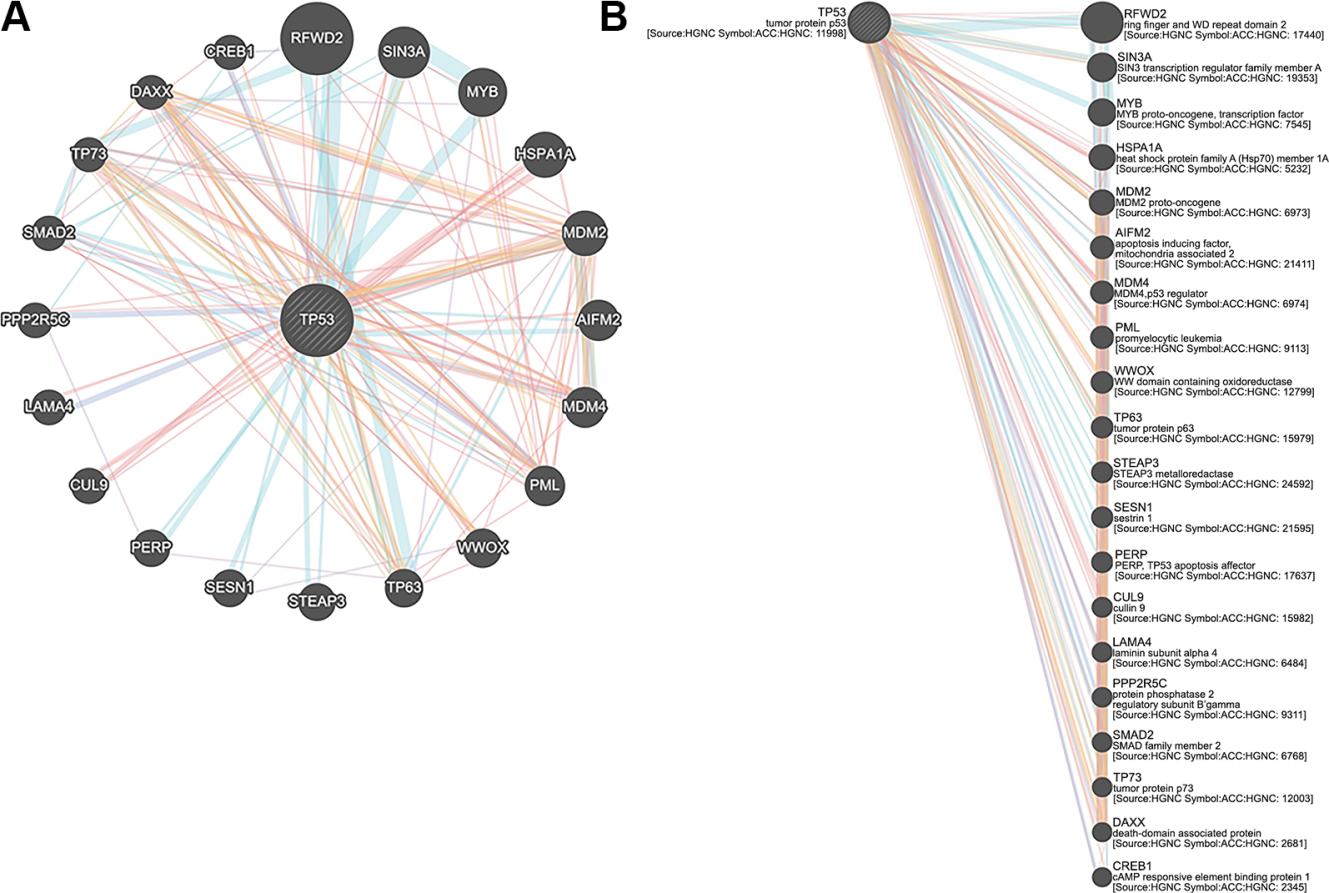
**(A)** Tumor protein 53 (TP53) network analysis and **(B)** members of the complex pathway and genes with co-expression, co-localization, genetic interactions and specific functions.

The exclusive over-expression of HHV-8 LANA-1 in KS confirms significant sensitivity and specificity. The domain is conserved in the HHV-8 and ORF-73, suggesting its expression during viral latency and allowing it to interact with p53, thereby inducing the apoptosis pathway. The evidence from another study indicates abnormal expression of p53 in the nodular region and metastatic lesion of angiosarcomas (rather than in the primary lesion) (Yee-Lin et al., 2018). To account for this, the lead p53 in KS was taken with reference to the database for a herpes virus-associated infection model so as to understand the immune evasion with a detailed pathway demonstrating the dominant role of a p53 oncogene in KSHV- (**Figure 5**). The tumor suppressor antigen p53 depends on cellular conditions inducing arrest of the cell growth and controlling cell division. This process inhibits cyclin-dependent kinases mediated by the expression of BAX and FAS antigens or by the repression of the Bcl-2expression (Kanashiro et al., 2003). Addressing the markers involved in the cell-cycle arrest is important to understand the molecular evolution of KS and for work towards its eradication. We examined PPIs to explore the complex biochemical interactions and molecular functions of proteins of interest with cellular components, as reported in **Table 3**. **Table 3** also presents the functional enrichment of p53 including its biological process, molecular functions, and cellular components. The effector p53 is directly involved in the arrest of the G1/S cell-cycle progression from normal to cancerous cells (Chen, 2016). Analysis of PPI with STRING showed an enriched p-value of 1.31e−05 with respect to the network having significantly more interactions than expected with 11 nodes, 47 edges, an average node degree of 8.55 and an average local cluster coefficient of 0.919 (**Figure 6**). The functions of the protein p53, a tumor protein, are associated with various expression levels during oncogenesis. GeneMania predicted various valuable functions of the query protein and interacting partners associated with it (**Figure 7**).

**Table 3:** Functional enrichment of p53.

Biological process (GO)				
Sl. No	GO-term	Description	Count in gene set	False discovery rate
1	GO:0016579	Protein deubiquitination	10 of 275	3.83e−15
2	GO:0007249	I-kappaB kinase/NF-kappaB signaling	8 of 70	3.83e−15
3	GO:0035666	TRIF-dependent toll-like receptor signaling pathway	6 of 24	8.43e−13
4	GO:0051092	Positive regulation of NF-kappaB transcription factor activity	5 of 2142	6.64e−11
5	GO:0070423	Nucleotide-binding oligomerization domain	5 of 27	4.65e−10
Molecular function (GO)				
1	GO:0031625	Ubiquitin protein ligase binding	5 of 311	4.44e−05
2	GO:0042975	Peroxisome proliferator activated receptor binding	2 of 10	0.00062
3	GO:0019899	Enzyme binding	7 of 2197	0.0012
4	GO:0042802	Identical protein binding	6 of 1754	0.0032
5	GO:0032813	Tumor necrosis factor receptor superfamily binding	2 of 46	0.0052
Cellular components (GO)				
1	GO:0043657	Host cell	4 of 29	2.76e−07
2	GO:0030666	Endocytic vesicle membrane	5 of 152	2.90e−07
3	GO:0098805	Whole membrane	8 of 1554	3.85e−06
4	GO:0012506	Vesicle membrane	6 of 743	1.69e−05
5	GO:0005741	Mitochondrial outer membrane	4 of 181	3.05e−05
KEGG pathway				
1	hsa04668	TNF signaling pathway	4 of 108	1.27e−05
2	hsa04064	NF-kappa B signaling pathway	4 of 93	1.27e−05
3	hsa05160	Hepatitis C	4 of 131	1.60e−05
4	hsa04210	Apoptosis	4 of 135	1.60e−05
5	hsa05167	Kaposi's sarcoma-associated herpesvirus infection	4 of 183	3.53e−05
Reactome pathways				
1	HSA-5357956	TNFR1-induced NFkappaB signaling pathway	9 of 30	3.98e−21
2	HSA-5357905	Regulation of TNFR1 signaling	9 of 32	3.98e−21
3	HSA-5689880	Ub-specific processing proteases	10 of 202	1.94e−17
4	HSA-6804757	Regulation of TP53 Degradation	7 of 35	2.30e−15
5	HSA-5675482	Regulation of necroptotic cell death	6 of 17	2.63e−14
UniPort keywords				
1	KW-0832	Ubl conjugation	9 of 2380	1.28e−05
2	KW-0013	ADP-ribosylation	4 of 100	1.28e−05
3	KW-1017	Isopeptide bond	7 of 1713	0.00017
4	KW-0945	Host–virus interaction	4 of 432	0.00094
5	KW-0963	Cytoplasm	9 of 4972	0.0015
PFAM Protein Domains				
1	PF14560	Ubiquitin-like domain	4 of 14	3.12e−09
2	PF11976	Ubiquitin-2 like Rad60 SUMO-like	4 of 21	6.44e−09
3	PF00240	Ubiquitin family	4 of 46	7.76e−08
4	PF02201	SWIB/MDM2 domain	2 of 5	2.86e−05
5	PF00641	Zn-finger in Ran binding protein and others	2 of 16	0.00017
INTERPRO Protein Domains and Features				
1	IPR019956	Ubiquitin	4 of 12	1.83e−09
2	IPR019954	Ubiquitin conserved site	4 of 10	1.83e−09
3	IPR000626	Ubiquitin domain	4 of 57	3.14e−07
4	IPR016495	p53 negative regulator Mdm2/Mdm4	2 of 2	1.46e−05
5	IPR029071	Ubiquitin-like domain superfamily	4 of 184	1.75e−05
SMART Protein Domains				
1	SM00213	Ubiquitin homologues	4 of 45	6.77e−08
2	SM00005	DEATH domain, found in proteins involved in cell death	2 of 27	0.00035
3	SM00184	Ring finger	3 of 308	0.0012

### Pulldown Strategy and Protein Interaction Prediction for Biomarker Selection

Pull-down assays serve as a complementary method to further validate the predicted interactions in a quantitative manner towards understanding their dissociation constants and relative bindings of proteins and their direct binding sites. However, this is beyond the scope of this study. We believe the following recommendations should be followed by researchers investigating transient protein interactions: First, determining the protein solubility is essential. If the prey protein is at a too-high concentration, it will not be sufficiently soluble. Second, shortening the time and adjusting buffer conditions of incubation help prevent prey protein degradation. Third, checking the prey protein with beads if bait protein is not bound should be done as a control. Fourth, conducting all assays at a constant temperature of 4 °C should be considered if a variation in Kd is found between repeated experiments.

The tumor suppressor antigen p53 depends on specific cellular conditions to induce arrest of cell growth and to control cell division (Pucci et al., 2000; Chen, 2016).

**Figure 8:**
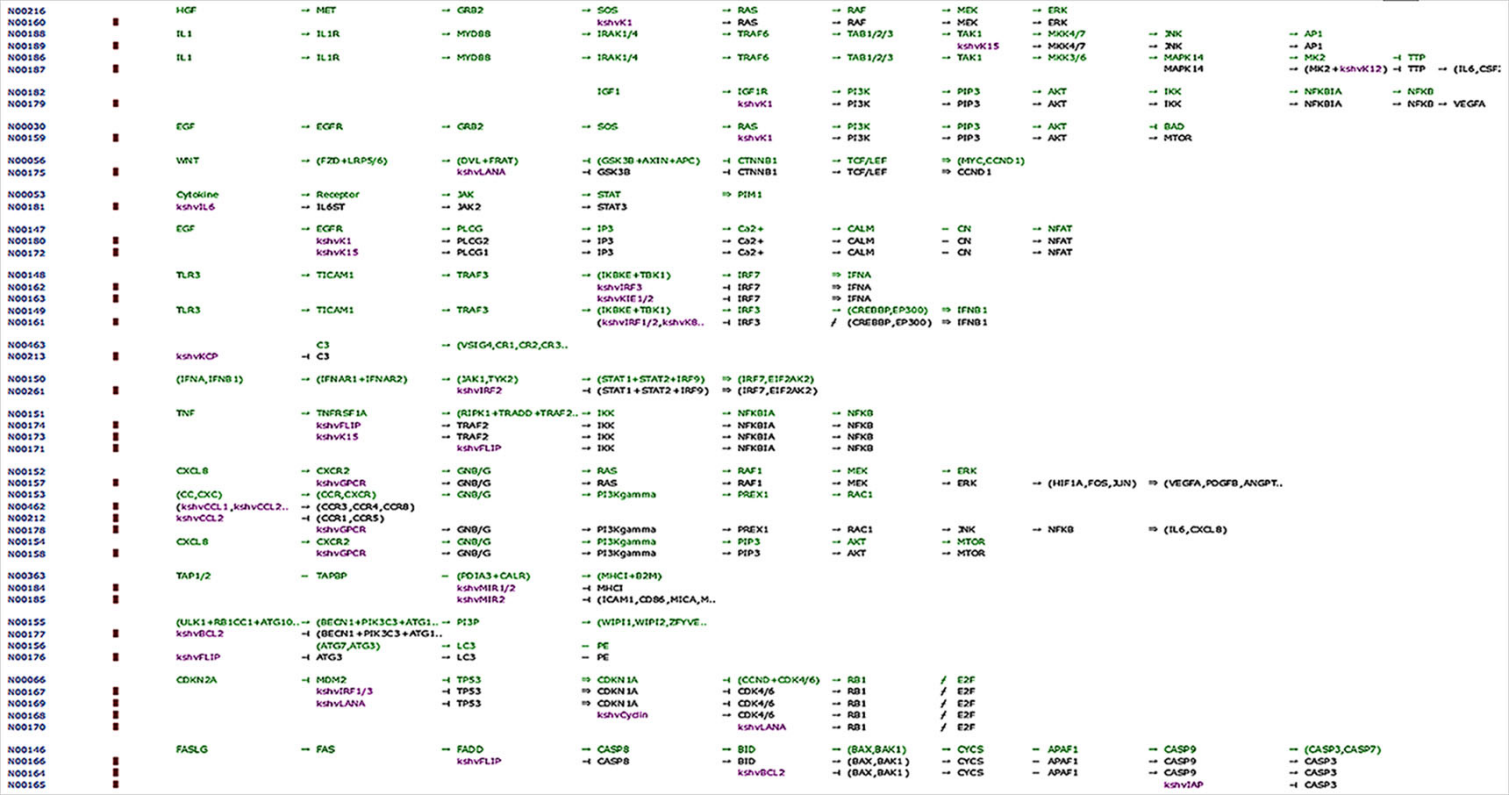
Network map of KEGG for the selected KS protein marker LANA. Protein downstream effect in the cell cycle of disease progression with pooled effectors in cell cycle arrest at G1/S and KS activating mechanisms.

Our network analysis (entry N00170, class nt06164) showed involvement of LANA and other effector markers in KS conditions and helped elucidate their mechanisms of action (**Figure 8**, **Table 4**). Therefore, we suggest that ORF-73 is an important protein that may be a useful biomarker for AIDS-related KS. Studies have suggested a linkage between ORF-73 and host apoptosis through p53 signaling pathways (Tornesello et al., 2018), that could represent a molecular mechanism for the predicted markers associated with KS. Our study discovered KS-associated markers which trigger cancer. ORF-73 encodes LANA-1 virtual proteins of KSHV, linking them with AIDS-associated KS, by their interaction with several cellular processes which include cell apoptosis (through p53) and inhibition of downstream transcriptomic performance. The association between HIV and ORF73 can be inferred by these findings.

**Table 4:** Identities of associated markers, downstream signaling candidates, and linked pathways during Kaposi sarcoma pathogenesis.

Sl. No	Entry	Description
1	N00216	HGF-MET-RAS-ERK signaling pathway
2	N00160	KSHV K1 to RAS-ERK signaling pathway
3	N00188	IL1-IL1R-JNK signaling pathway
4	N00189	KSHV K15 to JNK signaling pathway
5	N00186	IL1-IL1R-p38 signaling pathway
6	N00187	KSHV Kaposin B to p38 signaling pathway
7	N00182	IGF-IGFR-PI3K-NFKB signaling pathway
8	N00179	KSHV K1 to PI3K-NFKB signaling pathway
9	N00030	EGF-EGFR-RAS-PI3K signaling pathway
10	N00159	KSHV K1 to PI3K signaling pathway
11	N00056	Wnt signaling pathway
12	N00175	KSHV LANA to Wnt signaling pathway
13	N00053	Cytokine-Jak-STAT signaling pathway
14	N00181	KSHV vIL-6 to Jak-STAT signaling pathway
15	N00147	EGF-EGFR-PLCG-calcineurin signaling pathway
16	N00180	KSHV K1 to PLCG-calcineurin signaling pathway
17	N00172	KSHV K15 to PLCG-calcineurin signaling pathway
18	N00148	TLR3-IRF7 signaling pathway
19	N00162	KSHV vIRF3 to TLR3-IRF7 signaling pathway
20	N00163	KSHV KIE1/2 to TLR3-IRF7 signaling pathway
21	N00149	TLR3-IRF3 signaling pathway
22	N00161	KSHV vIRF1/2 to TLR3-IRF3 signaling pathway
23	N00463	Alternative pathway of complement activation
24	N00213	KSHV Kaposin to alternative pathway of complement activation
25	N00150	Type I IFN signaling pathway
26	N00261	KSHV vIRF2 to IFN signaling pathway
27	N00151	TNF-NFKB signaling pathway
28	N00174	KSHV vFLIP to TNF-NFKB signaling pathway
29	N00173	KSHV K15 to TNF-NFKB signaling pathway
30	N00171	KSHV vFLIP to NFKB signaling pathway
31	N00152	CXCR-GNB/G-ERK signaling pathway
32	N00157	KSHV vGPCR to GNB/G-ERK signaling pathway
33	N00153	CCR/CXCR-GNB/G-PI3K-RAC signaling pathway
34	N00462	KSHV vCCL1/2/3 to CCR signaling pathway
35	N00212	KSHV vCCL2 to CCR signaling pathway
36	N00178	KSHV vGPCR to GNB/G-PI3K-JNK signaling pathway
37	N00154	CXCR-GNB/G-PI3K-AKT signaling pathway
38	N00158	KSHV vGPCR to GNB/G-PI3K-AKT signaling pathway
39	N00363	Antigen processing and presentation by MHC class I molecules
40	N00184	KSHV MIR1/2 to antigen processing and presentation by MHC class I molecules
41	N00185	KSHV MIR2 to cell surface molecule-endocytosis
42	N00155	Autophagy-vesicle nucleation
43	N00177	KSHV vBCL2 to autophagy-vesicle nucleation
44	N00156	Autophagy-vesicle elongation
45	N00176	KSHV vFLIP to autophagy-vesicle elongation
46	N00066	MDM2-p21-Cell cycle G1/S
47	N00167	KSHV vIRF1/3 to p21-cell cycle G1/S
48	N00169	KSHV LANA to p21-cell cycle G1/S
49	N00168	KSHV vCyclin to cell cycle G1/S
50	N00170	KSHV LANA to cell cycle G1/S
51	N00146	Crosstalk between extrinsic and intrinsic apoptotic pathways
52	N00166	KSHV vFLIP to crosstalk between extrinsic and intrinsic apoptotic pathways
53	N00164	KSHV vBCL2 to crosstalk between extrinsic and intrinsic apoptotic pathways
54	N00165	KSHV vIAP to crosstalk between extrinsic and intrinsic apoptotic pathways

## Discussion

Many viral genes are homologous to host cellular genes in KSHV (Swanton et al., 1997). The PubMed, Google Scholar, and Scopus searches confirmed the key diagnostic markers for KS based on the available literature. Our computational study on them revealed their importance and evolutionary role in human cancer biology. LANA-1 imparts important immunogenic effects to KSHV, and it specifically interacts with many cellular pathways, including that of cell apoptosis (through its interaction with p53, and repression of downstream transcripts; see **Table 4**). This induces oncogenesis by targeting the protein-E2F transcriptional regulatory pathway (Radkov et al., 2000). The protein homologues identified through our search were structurally different from each other. Therefore, we analyzed selected proteins and compared them using homology searches for the selected domains to prove interactions with other host proteins that trigger and induce cancer in individuals with immunosuppression (Kersse et al., 2011). Hyper mutation and conserved structural sequence similarities help to maintain key aspects of secondary and tertiary structures, which were consistent with the computational analyses in our study (Huang et al., 2002). **Figure 5** shows the KSHV infection pathway from KEGG. We highlighted the reference pathway using a red box that shows that LANA is associated with the p53 signaling pathway. A BLAST homology search confirmed an ORF-73 marker interaction during herpesvirus pathogenesis. The results of STRING and KEGG searches suggested ORF-73 interacts with the host p53.

ORF-73 is not the only protein marker implicated in KS pathology, but much about it remains unknown. It is used as a marker for KSHV; especially, its protein folding and motifs are important for the marker assessment observed in the pattern of structural domains in the selected sequence analyzed with PSI-PRED. The pathogenic interactions in the network-based analysis between LANA and the host p53 suggest that LANA was confirmed by STRING and FUGUE tools. The predicted sequence motifs give detailed interactions that are conserved in the subfamilies of the herpesviruses as discussed in detail on the KEGG pathway with notable mechanisms described in the literature (Schulz, 2000; Direkze and Laman, 2004; Sharma-Walia et al., 2004; Mesri et al., 2010). However, the markers associated with KS need to be incorporated into comprehensive clinical cohort studies, designed using differential protein purification techniques and evidence-based knowledge on protein interactions with bait proteins to develop practical medical applications in the future.

Many PPIs have been elucidated using pull-down assays to map the genomes of many organisms, such as yeast (Valente et al., 2009), *Escherichia coli* (Arifuzzaman et al., 2006) *Caenorhabditis elegans* (Remmelzwaal and Boxem, 2019).

Like all other herpesviruses, KSHV displays latency and a lytic life cycle replication that are characteristic of some viral gene expressions. The genes LANA, v-FLIP, v-cyclin, and Kaposins A, B, and C for latency facilitate the establishment of life in its host and survival against host immune mechanisms. During latency, proteins expressed as K1, K15, vIL6, vGPCR, vIRFs, and vCCLs participate in inflammatory and angiogenic processes evident in KS lesions. Many other lytic and latent viral proteins are involved in the transformation of KSHV host cells into malignant cells. Also, Bcl-2 is one of the major KS progression factors, and TP53 and c-myc have a role in the progression of disease. KS pathology is interconnected with immune modulation effects such as cell cycle arrest in the host cell, which is required for pathogenic conditions and is mitigated by modulating key factors such as LANA.

Likewise, measuring the expression level and identifying the function of the encoded protein products is important to understand the pathogenesis of KS. We used a methodology similar to that in co-immunoprecipitation (Co-IP) experiments because of our ligand's affinity to capture the strongest interacting proteins (Lapetina and Gil-Henn, 2017). MS identifies subunits and helps explore the structural information associated with the protein of interest (Byrum et al., 2012). Dynamic PPI machines assemble or disassemble the ever-changing inter-, intra-, and extracellular influx cues as a preliminary step towards understanding the structure of proteins and to determine their functions to identify the relevant pathways of interacting proteins (Einarson, 2001; Vikis and Guan, 2004; Einarson et al., 2007). The role and important reason to select ORF-73 in the study is that encoding LANA protein distinct domain induces a putative nuclear localization signal (NLS), which product shown interacting with many co-cellular p53, pRb, and ATF4/CREB2. LANA also modulates transcriptional activity of HIV-1 long terminal repeat and to understand the how ORF-73 appears to prevent activity of KS-associated genes was new to know to make preventive strategy (Schäfer et al., 2003). Our findings may help researchers planning cancer prevention strategies, but we used common computational analyses alone, and future studies with expression and interaction analyses should be used to confirm our results and generate treatment options for KS.

## Conclusion

Our computational studies found that ORF-73 is involved in host apoptosis through p53 signaling pathways and is a key marker associated for Kaposi Sarcoma. This study also identified potential KS-associated genes which are reported to trigger cancer and suggested mechanisms of interaction that may help researcher developing prevention strategies.

## Ethics Statement

We retrieved all data from publicly available resources and we required no ethical approvals for dissemination of this purely academic information.

## Author Contributions

PZ, JW, and XZ conceived and designed the study. XW, LJ and XG provided study materials and were responsible for the collection and assembly of data, data analysis, and interpretation. PZ was involved in writing of the manuscript. All authors read and approved the final manuscript.

## Funding

This study was supported by the Seed Fund Program of Shanghai University of Medicine and Health Sciences (Grant No. SFP-18-21-01-002), the General Program of Pudong New Area Health and Family Planning Commission of Shanghai, China (Grant No. PW2016A-7), the National Natural Science Foundation of China (No. 81830052), Construction project of Shanghai Key Laboratory of Molecular Imaging (18DZ2260400), Shanghai Municipal Education Commission (Class II Plateau Disciplinary Construction Program for Medical Technology of SUMHS, 2018-2020), and the Natural Science Foundation of Guangdong Province (2016A030313680).

## Conflict of Interest

The authors declare that the research was conducted in the absence of any commercial or financial relationships that could be construed as a potential conflict of interest.

## Supplementary Material

Supplementary Figure 1 Flow chart showing the methodology for choosing selective markers for downstream analyses to develop a PPI network.
