# Supplementary figures and images for: Identification of AIDS-Associated Kaposi Sarcoma: A Functional Genomics Approach
Source: Front Genet. 2020 Jan 24;10:1376. doi: 10.3389/fgene.2019.01376 (PMC6992650; doi:10.3389/fgene.2019.01376)

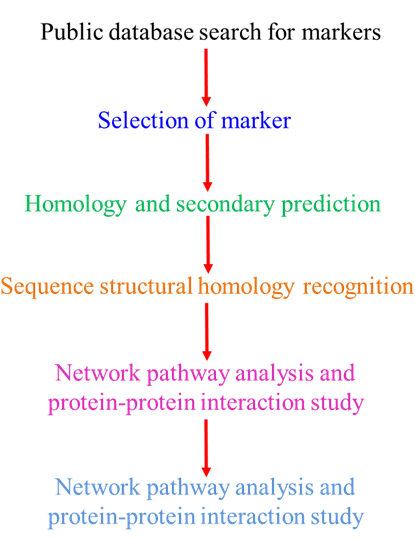

Supplement: Supplementary Figure 1 — Flow chart showing the methodology for choosing selective markers for downstream analyses to develop a PPI network. [file Image_1.tif]
